# Association of dietary decanoic acid intake with diabetes or prediabetes: an analysis from NHANES 2005–2016

**DOI:** 10.3389/fnut.2024.1483045

**Published:** 2025-01-07

**Authors:** Huangxin Zhu, Qingan Fu, Ruxin Chen, Linfei Luo, Miao Yu, Yue Zhou

**Affiliations:** ^1^Department of Cardiovascular Medicine, The Second Affiliated Hospital of Nanchang University, Nanchang, China; ^2^Department of Gastroenterology, The Second Affiliated Hospital of Nanchang University, Nanchang, China

**Keywords:** decanoic acid, diabetes, prediabetes, NHANES, dietary fatty acids

## Abstract

**Background:**

With the increasing prevalence of prediabetes and diabetes, exploring dietary factors associated with prediabetes and diabetes has become a global health research priority. This study aimed to assess the relationship between dietary decanoic acid (DDA) intake and the risk of diabetes and prediabetes.

**Methods:**

Data from the National Health and Nutrition Examination Survey (NHANES) 2005–2016 included 11,477 adult participants. DDA intake was assessed through two 24-h dietary recalls and participants were grouped according to the diagnostic criteria for diabetes and prediabetes. Multivariate regression models were applied to analyze the relationship between DDA intake and diabetes and prediabetes, with subgroup analyses conducted to explore potential interactions.

**Results:**

Dietary decanoic acid intake was significantly negatively associated with the risk of diabetes. In the fully adjusted model, each 1 g/day increase in DDA intake was associated with a 19% reduction in the odds of developing diabetes from prediabetes (OR = 0.81, 95% CI: 0.68–0.96, *p* = 0.015) and this negative association was more pronounced in individuals with higher education level (*P* for interaction = 0.006). Compared with the DDA intake ≤0.18 g/day, DDA intake >0.58 g/day is related to reduced risk of progression to diabetes in prediabetic patients. However, the relationship between DDA intake and the risk of prediabetes was not statistically significant in the fully adjusted model (OR = 0.95, 95% CI: 0.84–1.07, *p* = 0.404).

**Conclusion:**

This study found that higher DDA intake may be associated with lower prevalence of diabetes among prediabetic population, and high education level strengthen this relationship.

## Introduction

Diabetes has become a significant public health concern worldwide, with the incidence of diabetes continuing to rise globally (1–4). As of 2019, the number of people living with diabetes globally is estimated at 463 million, and is projected to increase to 578 million by 2030 and 700 million by 2045 (5). Recognized as the fifth highest cause of mortality globally (6), diabetes may lead to various chronic complications including cancer (7), depression (8), diabetic retinopathy (9), diabetic neuropathy (10) and cardiovascular diseases (11). These diseases greatly reduce the enjoyment of quality of an individual’s life with diabetes and even increase the risk of death, greatly contributing to the medical and economic stress on society (12). Prediabetes refers to the transitional phase before the onset of diabetes, which is an intermediate hyperglycemic state between normoglycemia and diabetes, featuring impaired fasting glucose (IFG) and/or impaired glucose tolerance (IGT) (13, 14). The number of prediabetic adults has been increasing in recent years and is projected to grow to 418 million by 2025 (15). A study by the American Diabetes Association (ADA) shows that nearly 70% of prediabetes eventually develops into diabetes. Some studies have demonstrated that the pathogenesis of diabetes and prediabetes is multifactorial (13, 16–18), involving genetic predisposition, lifestyle choice and environmental factors, among which diet habits stands out as a key modifiable risk factor (19, 20).

In more recent times, there has been increasing emphasis on the contribution of nutritional fatty acids in development and progression of diabetes mellitus (21, 22). Decanoic Acid is one of medium chain fatty acids (MCFAs) containing 10 carbon atoms, which natural sources are limited, typically found in milk fat, coconut oil and palm kernel oil (23, 24). Multiple studies suggest that decanoic acid may control the incidence of coronary artery disease and epilepsy (25–27). In comparison to long-chain fatty acids (LCFAs), decanoic acid has unique metabolic characteristics that may affect blood glucose homeostasis and insulin sensitivity (28). The biological mechanisms of the metabolic effects of decanoic acid are well known, but there are no relevant studies based large-scale population examining the association between dietary decanoic acid (DDA) intake and diabetes or prediabetes (29).

In this research, we sourced data on DDA, as well as individuals with diabetes and prediabetes, from the database named National Health and Nutrition Examination Survey (NHANES) 2005 to 2016. The objective was to investigate the potential link between DDA intake and the risk of developing prediabetes or diabetes among adult Americans.

## Methods

### Study design and population

#### Measurement of dietary decanoic acid

NHANES is a cross-sectional survey program conducted by the Centers for Disease Control and Prevention (CDC), aimed at evaluating to the health and nutritional condition of adults and children across the United States. The dietary panel of NHANES focuses on collecting information about participants’ diets, including an assessment of dietary intake through a 24-h dietary recall record. The US Department of Agriculture’s the Food and Nutrient Database for Dietary Studies (FNDDS) records the content of 64 nutrients/food ingredients in all foods/beverages, including decanoic acid. Therefore, based on the food and beverages consumed by participants within 24 h and the amount of decanoic acid they contain, DDA intake within 24 h can be calculated. Since 2002, NHANES has collected 24 h of dietary recall data over 2 days using the U.S. In our study, DDA intake was estimated as the average of two 24-h recall cycles of decanoic acid in food.

### Diabetes and prediabetes

According to the diagnostic criteria of ADA, diabetes was defined as fasting blood sugar (FBG) ≥ 126 mg/dL or glycosylated hemoglobin (HbA1c) level ≥ 6.5%, or a “Yes” answer to any of the following questions: “Have doctors ever told you that you have diabetes?,” “Are you currently using insulin?,” and “Are you currently using oral hypoglycemic medications?.” Prediabetes was defined as having an FBG of 100 mg/dL-125 mg/dL or a HbA1c of 5.7–6.4%, or answering “Yes” to the question, “Have you ever been told you have prediabetes?.” Those who are not diagnosed with diabetes or prediabetes are defined as normal.

### Potential covariates

Covariates considered included some of the following demographic characteristics, laboratory tests and questionnaires: age (years), gender (male and female), race (Mexican American, non-Hispanic White, Non-Hispanic Black, Other Hispanic, other race), education level (Below high school, High school, and College or above), poverty income ratio (PIR, low level: PIR < 1.30; middle level: 1.30 ≤ PIR < 3.50; and high level: ≥3.50), body mass index (BMI, Kg/m^2^), waist (cm), and systolic blood pressure (SBP, mmHg) and diastolic blood pressure (DBP, mmHg), FBG (mg/dL), HbA1c (%), alanine aminotransferase (ALT, U/L), aspartate aminotransferase (AST, U/L), serum creatinine (SCR, μmol/L), triglyceride (TG, mmol/L), total cholesterol (TC, mmol/L), high density lipoprotein cholesterol (HDL-C, mmol/L), low density lipoprotein cholesterol (LDL-C, mmol/L). According to the results of the questionnaire, smoking status can be categorized into never (smoked less than 100 cigarettes in life), former (smoked more than 100 cigarettes in life but has now quit.) and current smokers (smoked more than 100 cigarettes in life and now still smoking). Drinking status based on gender and alcohol consumption was categorized into mild drinker (consuming <2 drinks/day for females, <3 drinks/day for males), moderate drinker (consuming ≥2 drinks/day for females, ≥3 drinks/day for males), heavy drinker (consuming ≥3 drinks/day for females, ≥4 drinks/day for males), and non-drinker. The diagnosis of hypertension relies on blood pressure measurements and medical history inquiry: SBP ≥ 140 mmHg and/or DBP ≥ 90 mmHg, or “being told by doctors that you have hypertension” and “currently taking antihypertensive medication.” A person is considered to be suffering from cardiovascular disease if he or she answers “Yes” to any of the following questions: “Ever been told you have congestive heart failure?,” “Ever been told you have coronary heart disease?,” “Ever been told you have angina pectoris?,” “Ever been told you have myocardial infarction?” and “Ever been told you have a stroke?”.

### Statistical analysis

In the baseline data analysis, we described the data according to the diagnostic criteria by dividing them into normal, prediabetic and diabetic groups. The continuous variables were expressed as median and quartiles, and the categorical variables were expressed as number and percentage. Kruskal–Wallis *H* and Mann–Whitney *U* tests were used for multiple inter- and intra-group comparisons of continuous variables, while chi-square tests were used for comparisons between categorical variables. In order to further explore the role of DDA in people with different levels of abnormal glucose metabolism, we will, respectively, conduct multivariate regression analysis of DDA intake in diabetes and prediabetes, diabetes and normal people, as well as prediabetes and normal people. Three regression models adjusted for different covariates were constructed for each group in two-by-two comparisons and were used to analyze within-group differences in the role of DDA in each group. Model 1 adjusts for no variables. Model 2 was adjusted for gender, age, ALT, SCR, TG, TC, HDL-C, LDL-C. Model 3 was adjusted for gender, age, education level, PIR, BMI, waist, smoking status, drink status, hypertension, cardiovascular, ALT, SCR, TG, TC, HDL-C, LDL-C. The adjustment of models in each group is completely consistent for ensuring maximum reduction of bias. Restricted cubic spline (RCS) analysis was used to intuitively display the non-linear associations between DDA and diabetes or prediabetes. Stratified analysis and interaction tests were performed to evaluate the factors that might influence the correlation between DDA and diabetes or prediabetes. These tests considered several variables, including gender (male and female), age (<60 years and ≥60 years), race (non-Hispanic white and other race), educational levels (High school or below and College or above), PIR (<3.0 and ≥3.0), BMI (<28 kg/m^2^ and ≥28 kg/m^2^), smoking status (Never and Smoker), drinking status (Never and Drinker), hypertension (Yes and No), cardiovascular (Yes and No). The findings of this study are articulated through the use of odds ratios (OR) and their corresponding 95% confidence intervals (95%CI). The statistical analyses were conducted within the R programming language environment (version 4.3.2). In this context, a *p*-value of less than 0.05 for both tails of the distribution, was adopted as the threshold for statistical significance.

## Results

### Selection of study population

**Figure 1:**
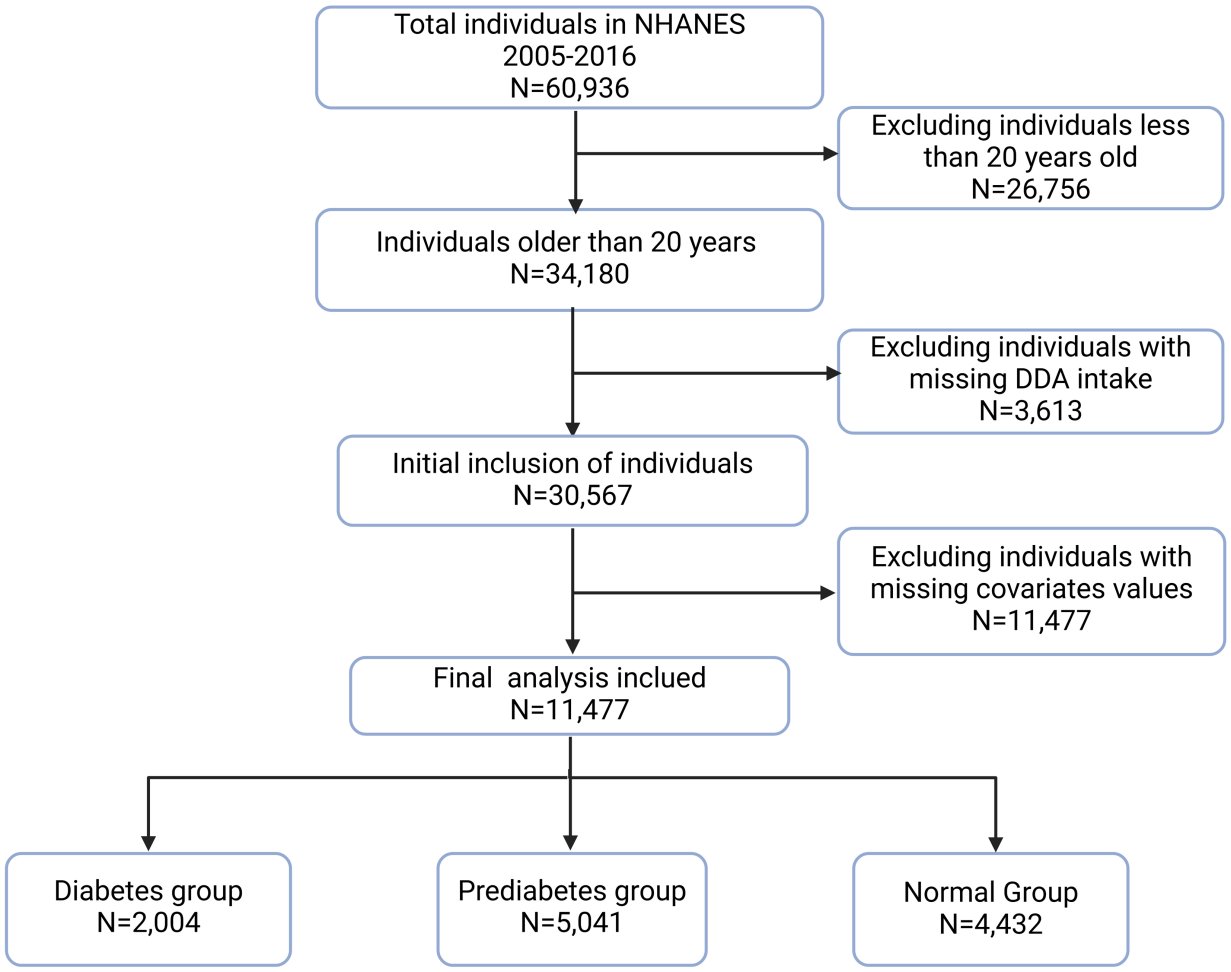
Flow chart of participants. DDA, dietary decanoic acid.

This study examined the relationship between DDA and diabetes or prediabetes using NHANES 2005–2016 data six survey cycles in total. These cycles recruited a total of 60,936 participants. Eligible participants are selected for analysis based on the following exclusion criteria: age < 20 years old (*N* = 26,756), missing DDA data (*N* = 3,613), and missing important covariates data (*N* = 19,090). Consequently, the final study population comprised 11,477 participants (Figure 1).

### Baseline characteristics of the study population

Table 1 showed the baseline characteristics of participants based on diabetes, prediabetes and normal population. This study included a total of eligible 11,477 individuals, among them, 2,004 (17.46%) suffered from diabetes, and 5,041 (43.92%) were in prediabetes state. In contrast to the normal population, individuals with diabetes or prediabetes tend to exhibit certain characteristics: they are often older, predominantly male, low-income, lower educated, smokers, non-drinkers, and non-Hispanic black ethnicity, and are more likely to experience cardiovascular and hypertension. Additionally, they have higher levels of BMI, waist, HbA1c, FBG, ALT, AST, SCR, TG, TC, LDL-C; and have lower HDL-C, DDA, total energy intake. Table 2 shows the baseline of participants based on quartiles of DDA intake. Contrasted with those in the lowest quartile, individuals in the top quartile of DDA intake are younger, male, non-Hispanic white, highly educated, high-income, non-hypertensive, non-cardiovascular, and non-diabetic. In addition, they have higher ALT and SCR levels and have lower HbA1c, FBG, and HDL levels.

**Table 1:** Weighted characteristics of participants with diabetes, prediabetes and normal from NHANES 2005 to 2016.

Variables	Overall	Normal	Prediabetes	Diabetes	*P*-value
	(*n* = 11,477)	(*n* = 4,432)	(*n* = 5,041)	(*n* = 2004)	
Age, years	49.00 (34.00,64.00)	37.00 (27.00,51.00)	53.00 (39.00,65.00)	62.00 (52.00,71.00)	<0.001
Gender (%)					<0.001
Male	5,647 (49.2)	1805 (40.7)	2,774 (55.0)	1,068 (53.3)	
Female	5,830 (50.8)	2,627 (59.3)	2,267 (45.0)	936 (46.7)	
Race (%)					<0.001
Mexican American	1782 (15.5)	619 (14.0)	813 (16.1)	350 (17.5)	
Non-Hispanic Black	2,240 (19.5)	769 (17.4)	969 (19.2)	502 (25.0)	
Non-Hispanic White	5,393 (47.0)	2,222 (50.1)	2,359 (46.8)	812 (40.5)	
Other Hispanic	1,063 (9.3)	394 (8.9)	459 (9.1)	210 (10.5)	
Other Race	999 (8.7)	428 (9.7)	441 (8.7)	130 (6.5)	
Education level (%)					<0.001
Below high school	2,737 (23.8)	824 (18.6)	1,248 (24.8)	665 (33.2)	
High school	2,643 (23.0)	916 (20.7)	1,222 (24.2)	505 (25.2)	
College or above	6,097 (53.1)	2,692 (60.7)	2,571 (51.0)	834 (41.6)	
PIR	2.18 (1.12,4.10)	2.31 (1.16,4.28)	2.19 (1.12,4.19)	1.91 (1.08,3.43)	<0.001
PIR (%)					<0.001
<1.3	3,515 (30.6)	1,307 (29.5)	1,542 (30.6)	666 (33.2)	
1.3–3.5	4,365 (38.0)	1,640 (37.0)	1877 (37.2)	848 (42.3)	
> = 3.5	3,597 (31.3)	1,485 (33.5)	1,622 (32.2)	490 (24.5)	
BMI, Kg/m^2^	27.90 (24.30,32.39)	25.91 (22.72,29.90)	28.40 (25.09,32.70)	31.20 (27.20,36.33)	<0.001
Waist, cm	97.90 (87.80,108.70)	91.10 (82.00,101.32)	99.60 (91.00,109.50)	107.70 (98.07,119.80)	<0.001
Smoking Status (%)					<0.001
Never	6,219 (54.2)	2,634 (59.4)	2,590 (51.4)	995 (49.7)	
Former	2,906 (25.3)	859 (19.4)	1,370 (27.2)	677 (33.8)	
Current	2,352 (20.5)	939 (21.2)	1,081 (21.4)	332 (16.6)	
Drink Status (%)					<0.001
Never	3,192 (27.8)	1,126 (25.4)	1,366 (27.1)	700 (34.9)	
Mild	7,820 (68.1)	3,129 (70.6)	3,451 (68.5)	1,240 (61.9)	
Moderate	219 (1.9)	86 (1.9)	109 (2.2)	24 (1.2)	
Heavy	246 (2.1)	91 (2.1)	115 (2.3)	40 (2.0)	
Cardiovascular (%)					<0.001
Yes	1,224 (10.7)	198 (4.5)	537 (10.7)	489 (24.4)	
No	10,253 (89.3)	4,234 (95.5)	4,504 (89.3)	1,515 (75.6)	
Hypertension (%)					<0.001
Yes	4,737 (41.3)	1,028 (23.2)	2,282 (45.3)	1,427 (71.2)	
No	6,740 (58.7)	3,404 (76.8)	2,759 (54.7)	577 (28.8)	
HbA1c, %	5.50 (5.20,5.90)	5.20 (5.00,5.40)	5.60 (5.40,5.80)	6.70 (6.10,7.70)	<0.001
FBG, mg/dL	100.00 (93.00,110.00)	92.00 (87.00,96.00)	104.00 (100.00,110.00)	134.00 (119.00,167.00)	<0.001
ALT, U/L	21.00 (16.00,28.00)	19.00 (15.00,26.00)	22.00 (17.00,29.00)	22.00 (17.00,31.00)	<0.001
AST, U/L	23.00 (19.00,27.00)	22.00 (19.00,26.00)	24.00 (20.00,28.00)	23.00 (20.00,28.00)	<0.001
SCR, μmol/L	74.26 (62.76,88.40)	70.72 (61.00,83.98)	76.91 (64.53,89.28)	77.79 (63.65,94.59)	<0.001
TG, mmol/L	1.17 (0.81,1.71)	1.02 (0.72,1.48)	1.22 (0.86,1.75)	1.41 (0.99,2.02)	<0.001
TC, mmol/L	4.91 (4.24,5.64)	4.86 (4.22,5.53)	5.04 (4.40,5.74)	4.63 (3.96,5.46)	<0.001
HDL-C, mmol/L	1.34 (1.11,1.63)	1.45 (1.19,1.73)	1.32 (1.09,1.58)	1.22 (1.03,1.50)	<0.001
LDL-C, mmol/L	2.87 (2.30,3.52)	2.82 (2.25,3.41)	3.03 (2.46,3.65)	2.64 (2.02,3.34)	<0.001
Total energy, kcal	1921.50 (1474.00,2477.50)	1969.50 (1516.50,2525.62)	1956.00 (1499.00,2507.50)	1750.75 (1323.50,2271.50)	<0.001
DDA, g/d	0.36 (0.19,0.59)	0.38 (0.20,0.61)	0.36 (0.19,0.59)	0.31 (0.16,0.53)	<0.001

The continuous variables were expressed as median and quartiles, and the categorical variables were expressed as number and percentage. PIR, poverty income ratio; BMI, body mass index; HbA1c, hemoglobin A1c; FBG, fasting blood glucose; ALT, alanine aminotransferase; AST, aspartate aminotransferase; SCR, serum creatinine; TG, triglyceride; TC, total cholesterol; HDL-C, high-density lipoprotein cholesterol; LDL-C, low-density lipoprotein cholesterol; DDA, dietary decanoic acid.

**Table 2:** Baseline characteristics of participants according to the quartiles of DDA intake.

Variables	Quartiles of DDA intake				*P*-value
	Q1 (≤0.192 g/d)	Q2 (0.192–0.359 g/d)	Q3 (0.359–0.594 g/d)	Q4 (>0.594 g/d)	
*N* (%)	2,901 (25.3)	2,904 (25.3)	2,890 (25.2)	2,782 (24.2)	
Age, years	52.00 (36.00, 66.00)	50.00 (35.00, 64.00)	48.00 (33.00, 63.00)	45.00 (32.00, 60.00)	<0.001
Gender (%)					<0.001
Male	1,303 (44.9)	1,285 (44.2)	1,406 (48.7)	1,653 (59.4)	
Female	1,598 (55.1)	1,619 (55.8)	1,484 (51.3)	1,129 (40.6)	
Race (%)					<0.001
Mexican American	520 (17.9)	474 (16.3)	430 (14.9)	358 (12.9)	
Non-Hispanic Black	743 (25.6)	611 (21.0)	482 (16.7)	404 (14.5)	
Non-Hispanic White	992 (34.2)	1,286 (44.3)	1,471 (50.9)	1,644 (59.1)	
Other Hispanic	323 (11.1)	261 (9.0)	266 (9.2)	213 (7.7)	
Other Race	323 (11.1)	272 (9.4)	241 (8.3)	163 (5.9)	
Education level (%)					<0.001
Below high school	905 (31.2)	717 (24.7)	604 (20.9)	511 (18.4)	
High school	695 (24.0)	692 (23.8)	637 (22.0)	619 (22.3)	
College or above	1,301 (44.8)	1,495 (51.5)	1,649 (57.1)	1,652 (59.4)	
PIR	1.90 (1.02, 3.63)	2.13 (1.12, 4.04)	2.35 (1.19, 4.29)	2.38 (1.20, 4.54)	<0.001
PIR (%)					<0.001
<1.3	1,016 (35.0)	896 (30.9)	816 (28.2)	787 (28.3)	
1.3–3.5	1,125 (38.8)	1,131 (38.9)	1,081 (37.4)	1,028 (37.0)	
> = 3.5	760 (26.2)	877 (30.2)	993 (34.4)	967 (34.8)	
BMI, Kg/m^2^	27.90 (24.30, 32.21)	28.00 (24.28, 32.63)	27.95 (24.30, 32.50)	27.80 (24.20, 32.17)	0.572
Waist, cm	97.50 (87.60, 107.80)	98.00 (87.50, 108.60)	97.75 (87.50, 109.20)	98.40 (88.50, 109.18)	0.107
Smoking status (%)					0.023
Never	1,610 (55.5)	1,576 (54.3)	1,596 (55.2)	1,437 (51.7)	
Former	707 (24.4)	751 (25.9)	732 (25.3)	716 (25.7)	
Current	584 (20.1)	577 (19.9)	562 (19.4)	629 (22.6)	
Drink status (%)					<0.001
Never	992 (34.2)	859 (29.6)	736 (25.5)	605 (21.7)	
Mild	1775 (61.2)	1928 (66.4)	2052 (71.0)	2065 (74.2)	
Moderate	59 (2.0)	64 (2.2)	51 (1.8)	45 (1.6)	
Heavy	75 (2.6)	53 (1.8)	51 (1.8)	67 (2.4)	
Cardiovascular (%)					<0.001
Yes	377 (13.0)	335 (11.5)	267 (9.2)	245 (8.8)	
No	2,524 (87.0)	2,569 (88.5)	2,623 (90.8)	2,537 (91.2)	
Hypertension (%)					<0.001
Yes	1,329 (45.8)	1,249 (43.0)	1,155 (40.0)	1,004 (36.1)	
No	1,572 (54.2)	1,655 (57.0)	1735 (60.0)	1778 (63.9)	
Diabetes (%)	602 (20.8)	539 (18.6)	448 (15.5)	415 (14.9)	<0.001
Prediabetes (%)	1,271 (43.8)	1,252 (43.1)	1,288 (44.6)	1,230 (44.2)	0.713
HbA1c, %	5.50 (5.20, 6.00)	5.50 (5.20, 5.90)	5.50 (5.20, 5.80)	5.50 (5.20, 5.80)	<0.001
FBG, mg/dL	100.00 (93.00, 111.00)	100.00 (93.00, 111.00)	100.00 (93.00, 109.00)	100.00 (93.00, 109.00)	0.009
ALT, U/L	21.00 (16.00, 28.00)	20.00 (16.00, 28.00)	21.00 (16.00, 28.00)	22.00 (17.00, 29.00)	<0.001
AST, U/L	23.00 (20.00, 28.00)	23.00 (19.00, 27.00)	23.00 (19.00, 27.00)	23.00 (19.00, 28.00)	0.057
SCR, μmol/L	73.37 (61.88, 88.40)	73.37 (61.88, 88.40)	74.26 (62.76, 88.40)	76.91 (65.42, 89.28)	<0.001
TG, mmol/L	1.16 (0.82, 1.71)	1.17 (0.82, 1.70)	1.19 (0.82, 1.75)	1.16 (0.80, 1.67)	0.656
TC, mmol/L	4.91 (4.22, 5.64)	4.91 (4.24, 5.61)	4.94 (4.27, 5.66)	4.86 (4.22, 5.61)	0.458
HDL-C, mmol/L	1.34 (1.11, 1.66)	1.34 (1.11, 1.63)	1.34 (1.11, 1.63)	1.32 (1.09, 1.60)	0.030
LDL-C, mmol/L	2.87 (2.30, 3.52)	2.87 (2.30, 3.49)	2.90 (2.30, 3.54)	2.87 (2.30, 3.52)	0.702
Total energy, kcal	1472.00 (1120.50, 1907.00)	1741.00 (1394.38, 2160.12)	2028.00 (1644.12, 2489.00)	2535.50 (2054.62, 3150.88)	<0.001

The continuous variables were expressed as median and quartiles, and the categorical variables were expressed as number and percentage. PIR, poverty income ratio; BMI, body mass index; HbA1c, hemoglobin A1c; FBG, fasting blood glucose; ALT, alanine aminotransferase; AST, aspartate aminotransferase; SCR, serum creatinine; TG, triglyceride; TC, total cholesterol; HDL-C, high-density lipoprotein cholesterol; LDL-C, low-density lipoprotein cholesterol; DDA, dietary decanoic acid.

### Association between DDA and diabetes or prediabetes

Table 3 presents a summary of the findings from the multiple logistic regression analysis.

**Table 3:** Association between DDA and odds of diabetes and prediabetes in different models.

DDA, g/d	Model 1		Model 2		Model 3	
	OR(95%CI)	*P*-value	OR(95%CI)	*P*-value	OR(95%CI)	*P*-value
Comparison between diabetes and prediabetes						
DDA	0.68(0.58,0.79)	<0.001	0.77(0.65,0.91)	0.002	0.81(0.68,0.96)	0.015
Q1(≤0.18)	Reference		Reference		Reference	
Q2(0.18–0.35)	0.92(0.80,1.06)	0.268	0.96(082,1.11)	0.571	0.98(0.84,1.14)	0.766
Q3(0.35–0.58)	0.74(0.64,0.86)	<0.001	0.77(0.66,0.90)	0.001	0.80(0.68,0.94)	0.006
Q4(>0.58)	0.70(0.61,0.81)	<0.001	0.79(0.68,0.92)	0.003	0.82(0.70,0.97)	0.019
P for trend	<0.001		<0.001		0.003	
Comparison between diabetes and normal						
DDA	0.60(0.51,0.70)	<0.001	0.76(0.63,0.92)	0.005	0.80(0.64,0.98)	0.035
Q1(≤0.19)	Reference		Reference		Reference	
Q2(0.19–0.36)	0.82(0.71,0.95)	0.008	0.95(0.79,1.14)	0.586	0.95(0.78,1.16)	0.628
Q3(0.36–0.59)	0.67(0.58,0.77)	<0.001	0.78(0.65,0.94)	0.009	0.82(0.67,1.00)	0.055
Q4(>0.59)	0.62(0.53,0.72)	<0.001	0.79(0.65,0.95)	0.013	0.82(0.67,1.01)	0.065
*P* for trend	<0.001		0.003		0.027	
Comparison between prediabetes and normal						
DDA	0.90(0.81,1.00)	0.057	0.94(0.83,1.06)	0.307	0.95(0.84,1.07)	0.404
Q1(≤0.20)	Reference		Reference		Reference	
Q2(0.20–0.37)	0.93(0.83,1.05)	0.226	0.95(0.84,1.08)	0.438	0.96(0.84,1.09)	0.503
Q3(0.37–0.60)	0.91(0.81,1.02)	0.089	0.95(0.84,1.08)	0.424	0.96(0.84,1.09)	0.521
Q4(>0.60)	0.88(0.79,0.99)	0.032	0.93(0.82,1.06)	0.275	0.94(0.82,1.07)	0.322
*P* for trend	0.029		0.297		0.353	

DDA, dietary decanoic acid. Model 1 adjusts for no variables. Model 2 adjusts for gender, age, ALT, SCR, TG, TC, HDL-C, LDL-C. Model 3 adjusts for gender, age, education level, PIR, BMI, waist, smoking status, drink status, cardiovascular, hypertension, ALT, SCR, TG, TC, HDL-C, LDL-C.

Comparison between diabetes and prediabetes, there is a negative association between DDA consumption and the prevalence of diabetes among prediabetic patients. In the unadjusted Model 1, for every additional 1 g/d of DDA intake, the OR for diabetes prevalence was reduced by 32% (OR = 0.68, 95%CI: 0.58–0.79, *p* < 0.001). This association persisted strongly even after accounting for potential confounding variables in the adjusted Model 2 (OR = 0.77, 95%CI: 0.65–0.91, *p* = 0.002) and Model 3 (OR = 0.81, 95%CI: 0.68–0.96, *p* = 0.015).

Comparison between diabetes and normal, DDA intake is also negatively correlated with the diabetes prevalence in normal people. In the Model 1, the OR for prevalence of diabetes was lowered by 40% with each 1 g/d increase in DDA intake (OR = 0.60, 95%CI: 0.51–0.70, *p* < 0.001). This trend continued to hold in the adjusted Model 2 (OR = 0.76, 95%CI: 0.63–0.92, *p* = 0.005) and Model 3 (OR = 0.80, 95%CI: 0.64–0.98, *p* = 0.035).

Comparison between prediabetes and normal, Model 1 indicated no significant relationship between DDA intake and prediabetes in the normal population (OR = 0.90, 95% CI: 0.81–1.00, *p* = 0.057). Despite adjustments for confounding factors in Model 2 (OR = 0.94, 95% CI: 0.83–1.06, *p* = 0.307) and Model 3 (OR = 0.95, 95% CI: 0.84–1.07, *p* = 0.404), the correlation did not reach statistical significance.

Multiple logistic regression analysis showed that DDA intake was significantly negatively correlated with the prevalence of diabetes whether in the normal or prediabetes people, but there was no correlation between DDA intake and the prevalence of prediabetes in normal people. These results suggest that DDA might primarily function to impede the transition from a prediabetic state to diabetes. Consequently, our subsequent research will concentrate on examining the impact of DDA intake on this progression from prediabetes to diabetes. In trend testing for DDA intake quartiles and diabetes among prediabetic patients in Table 3, comparing the second, third, and fourth quartiles of DDA intake to the lowest quartile, the OR and 95% CI for diabetes in the fully adjusted Model 3 were, respectively, 0.98 (0.84–1.14), 0.80 (0.68–0.94), and 0.82 (0.70–0.97), with a significant trend (*P* for trend = 0.003).

**Figure 2:**
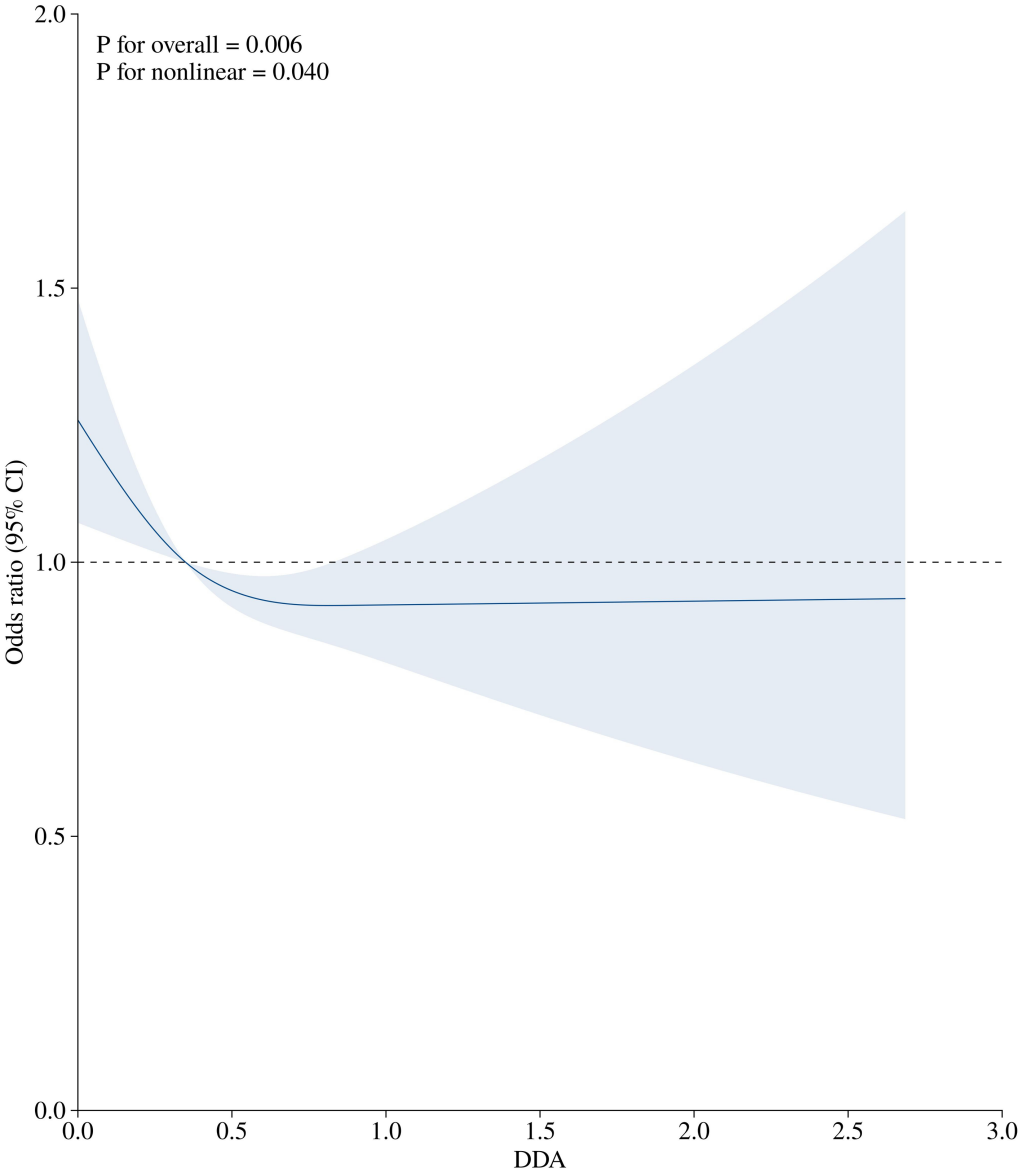
The association of DDA intake (g/d) with the prevalence of diabetes among prediabetic population. The OR (solid lines) and 95%CI (shaded areas) in the RCS was adjusted for gender, age, education level, PIR, BMI, waist, smoking status, drink status, cardiovascular, hypertension, ALT, SCR, TG, TC, HDL-C, LDL-C.

After multivariable adjustment in Model 3, a non-linear association was observed between DDA intake and the risk of diabetes among prediabetic population, as depicted in the RCS curve in Figure 2 (*P* for overall = 0.006; *P* for non-linear = 0.004). The RCS curves showing other relationships, including DDA intake with prediabetes and DDA intake with diabetes in normal population, can be found in the Supplementary material.

### Subgroup analyses

**Figure 3:**
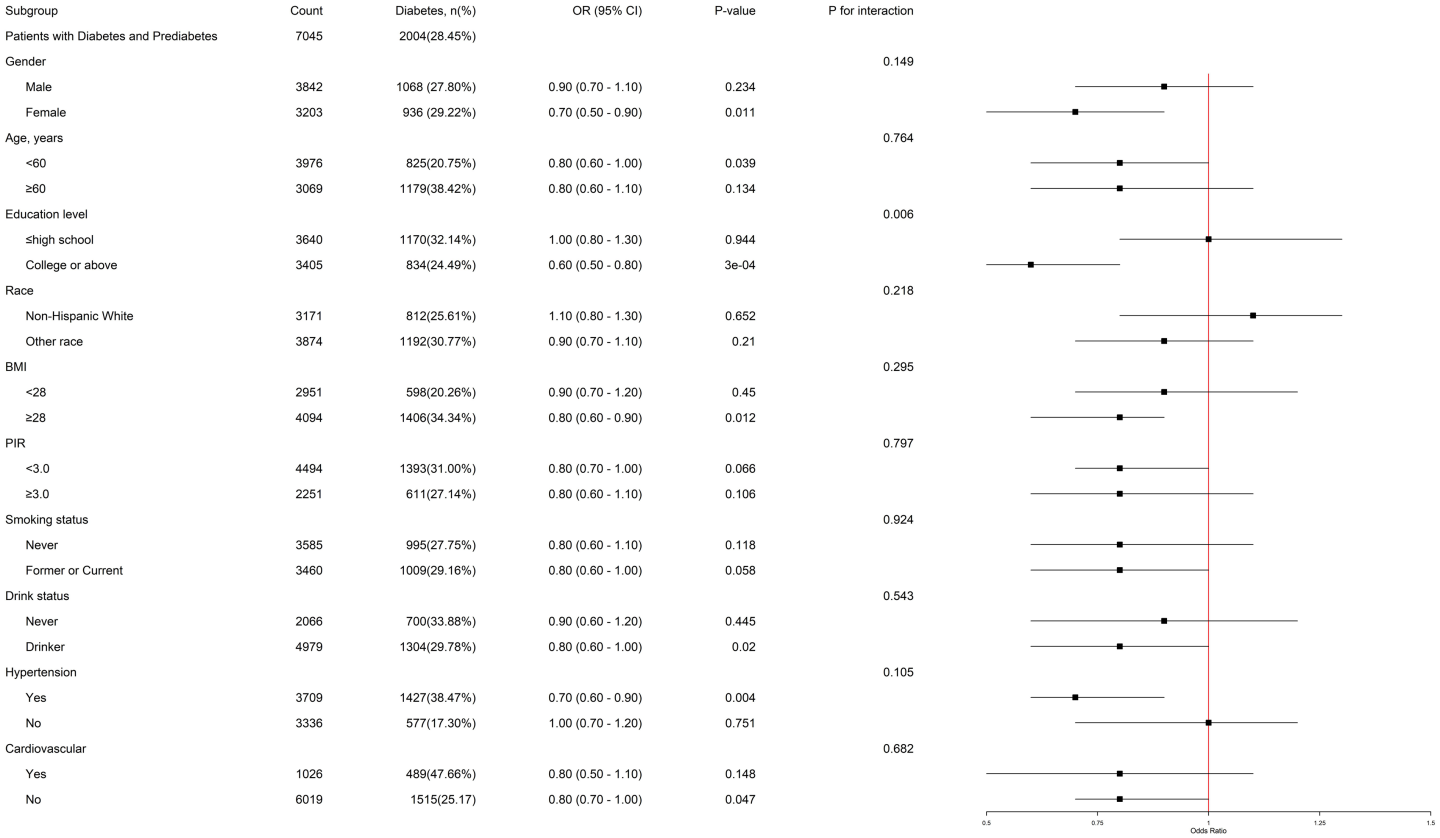
Subgroup analysis of the association of DDA intake and the risk of diabetes among prediabetic population. Each subgroup analysis was adjusted for gender, age, education level, PIR, BMI, waist, smoking status, drink status, cardiovascular, hypertension, ALT, SCR, TG, TC, HDL-C, and LDL-C. The strata variable was not included when stratifying by itself.

In subgroup analysis, among female individuals, people with education level of college or above, BMI ≥ 28 kg/m^2^, and hypertension, DDA has a more significantly negative correlation with the prevalence of diabetes in prediabetes patients. Notably, a significant interaction effect was identified between DDA intake and educational level in relation to the risk of diabetes in prediabetic individuals (*P* for interaction = 0.006) (Figure 3). This suggests that the impact of DDA intake on diabetic risk in prediabetes may vary depending on the education level.

## Discussion

Our study confirmed the negative correlation between DDA and diabetes for the first time based on large-scale population surveys.

As we all know, the development of diabetes is usually a gradual process, which generally goes through three stages: from normal blood sugar level to prediabetes, and finally to diabetes (14). As revealed by the multiple regression analysis and the RCS curve, the findings from this study demonstrate a significant negative relationship between DDA intake and the risk of diabetes among both prediabetic and normal individuals. However, no significant correlation was detected between DDA intake and the risk of prediabetes in the normal population. These inconsistent correlations implied that DDA potentially exerts a protective influence against the advancement of prediabetes rather than the onset of prediabetes. The results suggest that the role of DDA in lowering the prevalence of diabetes is primarily through its effect on slowing the transition from a prediabetic state to diabetes, rather than from a normal level to prediabetes. Compared with the DDA intake ≤0.18 g/day, DDA intake >0.58 g/day is related to reduced risk of progression to diabetes in prediabetic patients. Nevertheless, given prediabetic pivotal position in the progression to diabetes, the overall impact of DDA extends to reducing the risk of diabetes among normal individuals as well. This is because the preventive effect of DDA on the transition from prediabetes to diabetes indirectly lowers the overall prevalence of diabetes in the general population.

Prediabetes is the earlier stage before diabetes and eventually it contributed to the development of diabetes without effective treatment and control. About 5–10% of prediabetes patients become diabetes patients every year (30). Some studies show that 37% of prediabetes patients may develop diabetes within 4 years without timely treatment (31). Therefore, it is urgent to treat diabetes and prediabetes. Encouragingly, some studies have shown that the condition in the prediabetes stage is reversible, which provides a potential way for fighting against diabetes. Lifestyle intervention, pharmacological intervention and bariatric surgery are all important measures to prevent developing from prediabetes stage into diabetes (13, 32, 33). Lifestyle interventions stand out as the more rational and safer approach when juxtaposed with other two methods (32). Extensive longitudinal research has substantiated that adopting lifestyle modifications can notably extend the timeline before the transition from a prediabetic state to diabetes, with benefits observed over a decade (31). Regular and nutritious diet is also an important segment of lifestyle intervention. A plethora of research indicates that adhering to a diet rich in nutritious elements, particularly those with a low glycemic index like cereal fiber, whole grains, and bran, can lead to a decrease in diabetes risk by 18–40% (34). Additionally, the intakes of sugary drink has been shown to have a substantial impact on diabetes. Specifically, the risk of diabetes development escalates by 26% for individuals who regularly consume over one cup of sugar-sweetened drinks per day, in contrast to those who partake in less than one cup monthly (35, 36). These all highlights the significance of dietary habits in mitigating the risk of diabetes.

For over a decade, the role of MCFAs as a dietary component of ketogenic diet in regulating glucose and lipid metabolism has gradually become known (28). Ketogenic diet is a high-fat, moderate protein, and low carbohydrate diet that simulates the metabolic pattern of the body in a state of hunger by promoting the metabolism of fat in the body to produce ketone bodies (KBs) (37). The ingested medium chain triglycerides (MCT) are broken down into glycerol and MCFAs in the stomach and duodenum. Relying on the hydrophilicity and short carbon chain of MCFAs, they are allowed for a direct transportation via the portal vein to the liver and enter directly the mitochondria no need for the carnitine system (23). This enables a swift *β*-oxidation process to rapidly produce energy and MCFAs are converted into KBs, which may save consumption of muscle glycogen and liver glycogen, and reduce insulin demand (38). Under low-carbon or sugar free conditions, KBs produced by decanoic acid metabolism can serve as an energy source for the brain and other tissues, especially when glucose supply is limited (39).

The results in our study showed that an increased intake of DDA is linked to a reduced incidence of diabetes among the studied population. However, the underlying mechanism this correlation remains to be fully elucidated. It is likely that because decanoic acid plays a crucial role in alleviating insulin resistance (IR) and inflammatory response, which are key factors in the onset and progress of diabetes (18, 40). IR is the common pathway of prediabetes and diabetes, usually occurring in the years before diabetes or even prediabetes, and existing in the whole process from prediabetes to late diabetes (14, 18). An animal study based on male mice found that decanoic acid intake can effectively prevent obesity and promote glucagon-like peptide-1 (GLP-1) secretion through the MCFA receptor GPR84 to enhance glucose tolerance and improve insulin resistance (41). Abe et al. (42) found that decanoic acid enhances fatty acid oxidation capacity in mice without suppressing glycolysis in skeletal muscle. This effect is achieved by activating the peroxisome proliferator-activated receptor-*δ* (PPAR-δ), which in turn upregulates the expression of uncoupling protein 3 (UCP3) in skeletal muscle. Notably, this process does not interfere with the insulin-mediated phosphorylation of Akt, thus maintaining insulin signaling integrity.

Inflammation is a critical factor in the development of both diabetes and prediabetes (18, 43). The findings of a prospective study lasting 177 months in Rotterdam shown, after adjusting for multiple confounding factors, interleukin 13 (IL-13), extracellular newly identified receptor for advanced glycation end-products binding protein (EN-RAGE) and C-reactive protein (CRP) are still associated with prediabetes. In addition, certain inflammatory biomarkers, including adiponectin, CRP, and interleukin 6 (IL-6), have been correlated with the transition from a prediabetic state to diabetes (44). IL-6 and TNF-*α*, both pro-inflammatory cytokines involved in inflammation and immune responses, have some effects on regulating metabolism. IL-6 has been shown to increase IR and induce fasting hyperglycemia by stimulating glucagon release (45). TNF-α may increase IR through the promotion of phosphorylation of insulin receptor substrate-1 (IRS-1) (46). A study conducted both *in vitro* and *in vivo* using a acne murine model have demonstrated that capric acid (C10), the decanoic acid counterpart, exerts anti-inflammatory properties. This effect is achieved by curbing the phosphorylation of mitogen-activated protein kinase (MAPK) and the activation of nuclear factor kappa-B (NF-κB), thereby reducing the secretion of IL-6, IL-8 and TNF-*α* (47). In an animal experiment, it was observed that the supplementation of decanoic acid in the diet for miniature pigs led to a decrease in the levels of TNF-α and IL-6, attributed to the suppression of inflammatory gene expression (48). Peroxisome proliferator-activated receptor-*γ* (PPAR-γ) is a transcription factor that has anti-inflammatory properties and regulates mitochondrial function. The genetic variation and change of its gene expression may lead to mitochondrial dysfunction, which plays a role in the pathogenesis of IR (49, 50). As a direct ligand of PPAR-γ, decanoic acid can bind and partially activate PPAR-γ, stimulating mitochondrial biogenesis and increasing mitochondrial complex I activity to enhance mitochondrial function and improve glucose sensitivity (50–52). Although higher DDA intake can reduce the prevalence of diabetes and not prediabetes, we still recommend that they all need to increase the intake of decanoic acid, whether in people with normal blood glucose level or prediabetes. Because most prediabetes patients do not know their current status, these people can also slow down the progress to diabetes by supplementing appropriate decanoic acid.

As we discussed, decanoic acid, as one of MCFAs, has shown a positive effect in reducing the risk of diabetes. Other fatty acids also play different roles in the management of diabetes. The impact of fatty acids on metabolism varies with the length and saturation of the carbon chain. LCFAs have longer carbon chains compared to MCFAs. LCFAs such as palmitic acid may trigger insulin resistance in pancreatic beta cells by activating c-Jun N-terminal kinase expression (53). Excessive intake of LCFAs can cause lipid accumulation in the body, leading to lipotoxicity and insulin resistance (54). Long chain unsaturated fatty acids may promote the secretion of GLP-1 by activating the expression of G protein coupled receptors 120 (GPR120), thereby increasing circulating insulin (55).

The results of stratified analysis showed that among people with education level of college or above, DDA was still negatively correlated with the prevalence of diabetes in prediabetes patients, and there was interaction. Education level is an unchangeable social risk factor (56). A survey of educational differences among people with diabetes from 232 Latin American cities showed that there was a negative dose–response relationship between educational level and diabetes prevalence (57). In addition, genetic evidence suggests that higher levels of genetic decision education are associated with lower risk of type 2 diabetes (58). Individuals with higher levels of education are usually able to effectively access and understand the latest health research information, and tend to adopt healthy lifestyles, such as regular exercise and a healthy diet, which helps maintain a healthy weight and insulin sensitivity. They may have a deeper understanding of healthy diet and dietary fatty acids, including knowledge of different types of fatty acids in their diet. They may be more inclined to choose a diet containing healthy fats, which may indirectly affect the intake and metabolism of decanoic acid and reduce the risk of diabetes.

This study has some obvious advantages. Firstly, this study used data from NHANES 2005–2016, covering 6 cycles for a total of 12 years. This is a nationally representative large-scale sample database that ensures the accuracy of research results. Secondly, we established multiple models and adjusted for various potential confounding factors in order to reduce interference from other factors. In addition, we studied the relationship between DDA and people with different blood glucose status to ensure the reliability of the study. However, there are also some limitations that cannot be ignored. In the study, the diagnosis of patients with diabetes and prediabetes partly depends on the patient’s self-report, and dietary data was obtained through two 24-h dietary recalls, which may introduce memory bias to affect accuracy. This study adopts a cross-sectional design. We found that there is a negative correlation between DDA and the increased risk of diabetes, but it is difficult to determine the chronological order or causal relationship between them. Although the study has adjusted for multiple potential confounding factors, there may still be other unknown confounding factors that may affect the accurate assessment of true correlations. This study is based on American adults, and the conclusions drawn may not be applicable to populations in other countries.

## Conclusion

Our study found that higher DDA intake was associated with lower prevalence of diabetes among prediabetic patients, suggesting that DDA is a protective factor for diabetes. In the population with high education level, this relationship still holds and there is interaction.

## Data Availability

Publicly available datasets were analyzed in this study. This data can be found: https://www.cdc.gov/nchs/nhanes/index.htm.
