# Supplementary material for: Association of dietary decanoic acid intake with diabetes or prediabetes: an analysis from NHANES 2005–2016
Source: Front Nutr. 2025 Jan 7;11:1483045. doi: 10.3389/fnut.2024.1483045 (PMC11747714; doi:10.3389/fnut.2024.1483045)
Supplement: Supplementary file 1 [file Data_Sheet_1.docx]

Associations of DDA with diabetes among prediabetic population in various subgroups.

| Subgroups | Count | Diabetes, n(%) | OR(95%CI) | P-value | P for interaction |
| --- | --- | --- | --- | --- | --- |
| Patients with Diabetes and Prediabetes | 7045 | 2004(28.45%) |  |  |  |
| Gender |  |  |  |  | 0.149 |
| Male | 3842 | 1068 (27.80%) | 0.9(0.7,1.1) | 0.234 |  |
| Female | 3203 | 936 (29.22%) | 0.7(0.5,0.9) | 0.011 |  |
| Age, years |  |  |  |  | 0.764 |
| <60 | 3976 | 825(20.75%) | 0.8(0.6,1.0) | 0.039 |  |
| ≥60 | 3069 | 1179(38.42%) | 0.8(0.6,1.1) | 0.134 |  |
| Education level |  |  |  |  | 0.006 |
| High school or below | 3640 | 1170(32.14%) | 1.0(0.8,1.3) | 0.944 |  |
| College or above | 3405 | 834(24.49%) | 0.6(0.5,0.8) | 0.0003 |  |
| Race |  |  |  |  | 0.218 |
| Non-Hispanic White | 3171 | 812(25.61%) | 1.1(0.8,1.3) | 0.652 |  |
| Other race | 3874 | 1192(30.77%) | 0.9(0.7,1.1) | 0.210 |  |
| BMI, kg/m^2^ |  |  |  |  | 0.295 |
| <28 | 2951 | 598(20.26%) | 0.9(0.7,1.2) | 0.450 |  |
| ≥28 | 4094 | 1406(34.34%) | 0.8(0.6,0.9) | 0.012 |  |
| PIR |  |  |  |  | 0.797 |
| <3.0 | 4494 | 1393(31.00%) | 0.8(0.7,1.0) | 0.066 |  |
| ≥3.0 | 2251 | 611(27.14%) | 0.8(0.6,1.1) | 0.106 |  |
| Smoking status |  |  |  |  | 0.924 |
| Never | 3585 | 995(27.75%) | 0.8(0.6,1.1) | 0.118 |  |
| Smoker | 3460 | 1009(29.16%) | 0.8(0.6,1.0) | 0.058 |  |
| Drink status |  |  |  |  | 0.543 |
| Never | 2066 | 700(33.88%) | 0.9(0.6,1.2) | 0.445 |  |
| Drinker | 4979 | 1304(29.78%) | 0.8(0.6,1.0) | 0.020 |  |
| Hypertension |  |  |  |  | 0.105 |
| Yes | 3709 | 1427(38.47%) | 0.7(0.6,0.9) | 0.004 |  |
| No | 3336 | 577(17.30%) | 1.0(0.7,1.2) | 0.751 |  |
| Cardiovascular |  |  |  |  | 0.682 |
| Yes | 1026 | 489(47.66%) | 0.8(0.5,1.1) | 0.148 |  |
| No | 6019 | 1515(25.17) | 0.8(0.7,1.0) | 0.047 |  |

Abbreviations: DDA, dietary decanoic acid. Each subgroup analysis was adjusted for gender, age, education level, PIR, BMI, waist, smoking status, drink status, cardiovascular, hypertension, ALT, SCR, TG, TC, HDL-C, LDL-C. The strata variable was not included when stratifying by itself.

| 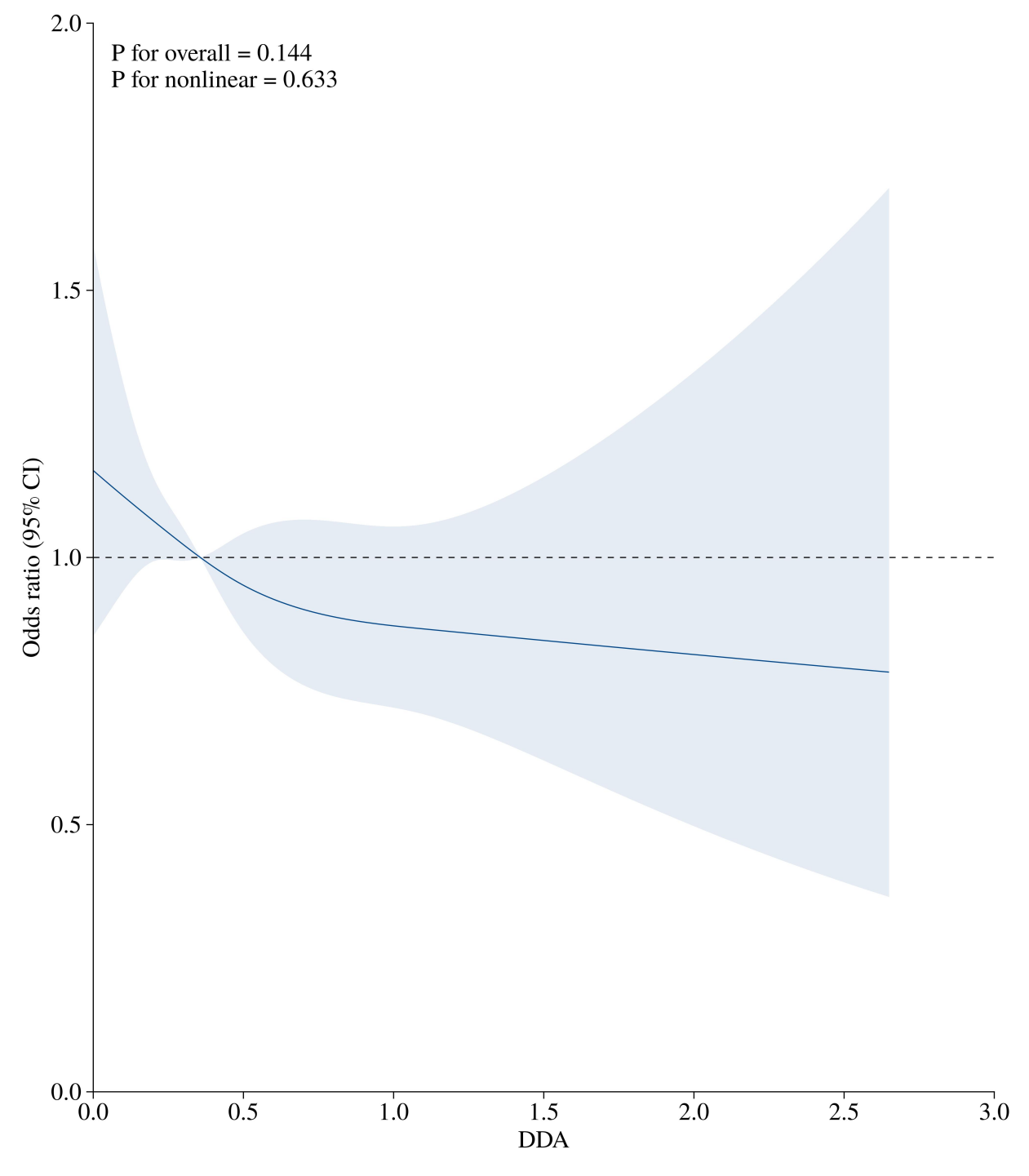 |
| --- |
| The association of DDA intake (g/d) with the prevalence of diabetes among normal population. The OR (solid lines) and 95%CI (shaded areas) in the RCS was adjusted for gender, age, education level, PIR, BMI, waist, smoking status, drink status, cardiovascular, hypertension, ALT, SCR, TG, TC, HDL-C, LDL-C. |

| 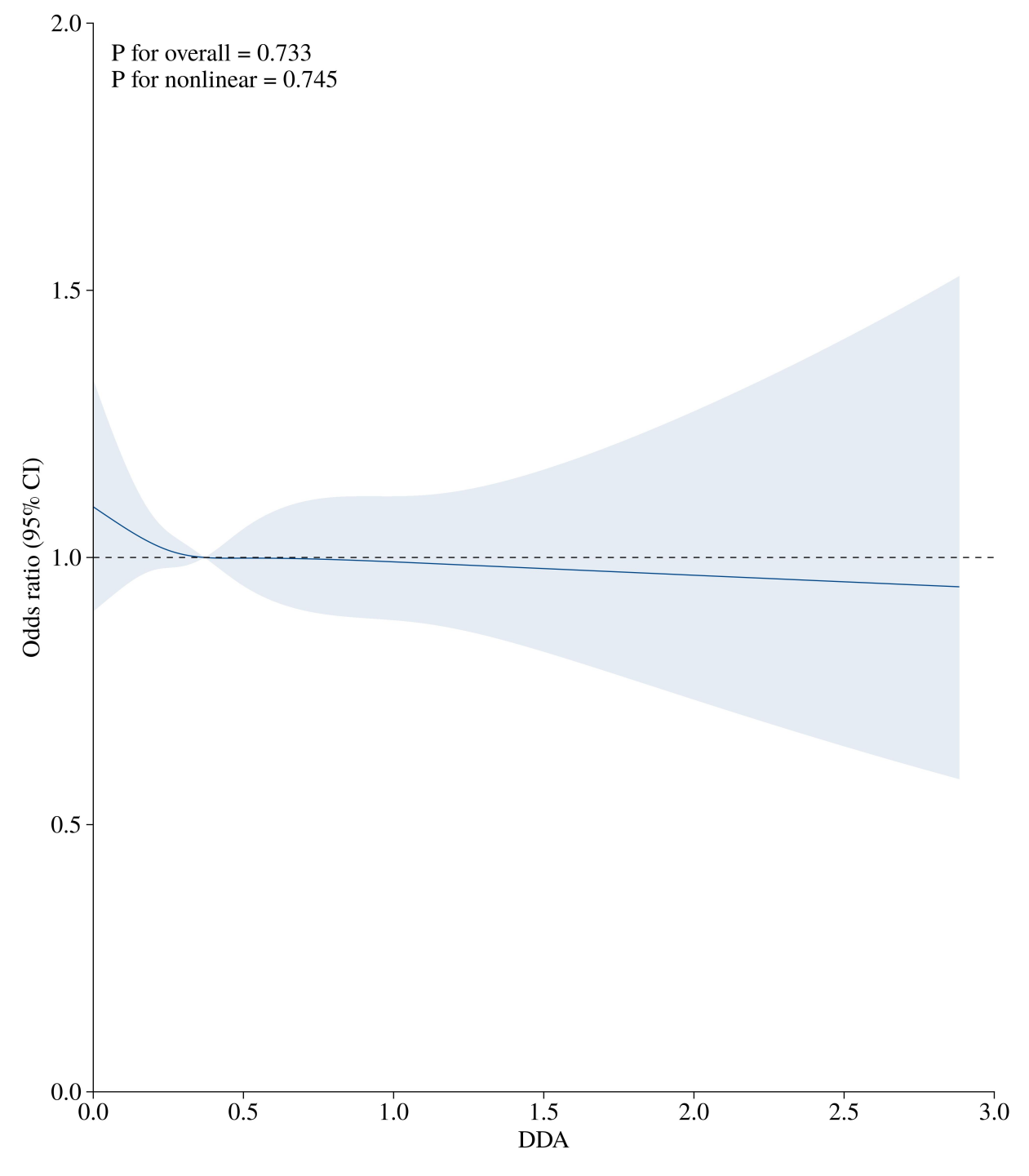 |
| --- |
| The association of DDA intake (g/d) with the prevalence of prediabetes among normal population. The OR (solid lines) and 95%CI (shaded areas) in the RCS was adjusted for gender, age, education level, PIR, BMI, waist, smoking status, drink status, cardiovascular, hypertension, ALT, SCR, TG, TC, HDL-C, LDL-C. |
